# Evaluation of the prescribing practice of guideline-directed medical therapy among ambulatory chronic heart failure patients

**DOI:** 10.1186/s12872-021-01868-z

**Published:** 2021-02-18

**Authors:** Daya Ram Parajuli, Sepehr Shakib, Joanne Eng-Frost, Ross A. McKinnon, Gillian E. Caughey, Dean Whitehead

**Affiliations:** 1 College of Nursing and Health Sciences, Flinders University, Adelaide, SA Australia; 2 Department of Clinical Pharmacology, Royal Adelaide Hospital, Adelaide, SA Australia; 3 Discipline of Pharmacology, Adelaide Medical School, University of Adelaide, Adelaide, SA Australia; 4 Department of Medicine, Royal Adelaide Hospital, Adelaide, SA Australia; 5 Department of Cardiology, Flinders Medical Centre, Adelaide, SA Australia; 6 Registry of Senior Australians, South Australian Health and Medical Research Institute, Adelaide, SA Australia; 7 Flinders Health and Medical Research Institute, Flinders University, Adelaide, SA Australia; 8 Flinders Rural Health, College of Medicine and Public Health, Flinders University, Ral Ral Avenue, PO Box 852, Renmark, SA 5341 Australia; 9 College of Health and Medicine, University of Tasmania, Tasmania, Australia

**Keywords:** Heart failure, Ejection fraction, Guideline-directed medical therapy, Pharmacist, Multidisciplinary, Comorbidities

## Abstract

**Background:**

Studies have demonstrated that heart failure (HF) patients who receive direct pharmacist input as part of multidisciplinary care have better clinical outcomes. This study evaluated/compared the difference in prescribing practices of guideline-directed medical therapy (GDMT) for chronic HF patients between two multidisciplinary clinics—with and without the direct involvement of a pharmacist.

**Methods:**

A retrospective audit of chronic HF patients, presenting to two multidisciplinary outpatient clinics between March 2005 and January 2017, was performed; a Multidisciplinary Ambulatory Consulting Service (MACS) with an integrated pharmacist model of care and a General Cardiology Heart Failure Service (GCHFS) clinic, without the active involvement of a pharmacist.

**Results:**

MACS clinic patients were significantly older (80 vs. 73 years, *p* < .001), more likely to be female (*p* < .001), and had significantly higher systolic (123 vs. 112 mmHg, *p* < .001) and diastolic (67 vs. 60 mmHg, *p* < .05) blood pressures compared to the GCHF clinic patients. Moreover, the MACS clinic patients showed more polypharmacy and higher prevalence of multiple comorbidities. Both clinics had similar prescribing rates of GDMT and achieved maximal tolerated doses of angiotensin-converting enzyme inhibitors (ACEIs) and angiotensin receptor blockers (ARBs) in HFrEF. However, HFpEF patients in the MACS clinic were significantly more likely to be prescribed ACEIs/ARBs (70.5% vs. 56.2%, *p* = 0.0314) than the GCHFS patients. Patients with both HFrEF and HFpEF (MACS clinic) were significantly less likely to be prescribed β-blockers and mineralocorticoid receptor antagonists. Use of digoxin in chronic atrial fibrillation (AF) in MACS clinic was significantly higher in HFrEF patients (82.5% vs. 58.5%, *p* = 0.004), but the number of people anticoagulated in presence of AF (27.1% vs. 48.0%, *p* = 0.002) and prescribed diuretics (84.0% vs. 94.5%, *p* = 0.022) were significantly lower in HFpEF patients attending the MACS clinic. Age, heart rate, systolic blood pressure (SBP), anemia, chronic renal failure, and other comorbidities were the main significant predictors of utilization of GDMT in a multivariate binary logistic regression.

**Conclusions:**

Lower prescription rates of some medications in the pharmacist-involved multidisciplinary team were found. Careful consideration of demographic and clinical characteristics, contraindications for use of medications, polypharmacy, and underlying comorbidities is necessary to achieve best practice.

## Background

Heart failure (HF) is a significant public health burden globally affecting an estimated 64 million people diagnosed worldwide. It has high rates of mortality and morbidity [1] with resultant impairment of quality of life [2]. Additionally, HF carries a significant financial burden on health care due to HF hospitalizations which are often prolonged [3] and also high rates of readmission within 30 days [4]. It is a complex syndrome driven by multiple comorbidities [5] and polypharmacy [6] due to its prevalence in the elderly population [7].

The European Society of Cardiology classification of HF is based on ejection fraction—with HFrEF defined as left ventricular ejection fraction (LVEF) < 40%, LVEF 40–49% for HFmrEF and LVEF > 50% for HFpEF [8]. HFpEF is an emerging area of clinical interest due to its increasing prevalence and changing risk factors which now includes increasing rates of hypertension [9]—markedly so in the female population [10]. HFpEF accounts for at least 50% of all diagnosis [11]. Furthermore, the emergence of HFmrEF presents an interesting development in HF given the results of a meta-analysis demonstrating its shared characteristics with both the HFrEF and HFpEF populations [12]. At present, HFmrEF remains a poorly understood phenotype due to the lack of randomized controlled trials included in this particular HF category [13].

While there is significant evidence for HFrEF management, there is a lack of evidence-based pharmacotherapies for HFpEF [8]. Guideline-recommended pharmacotherapies for HFrEF such as ACEIs/ARBs [14, 15], β-blockers [16, 17], and MRAs [18] have limited benefits in HFpEF, [19] and HFmrEF [20], likely reflecting the different underlying pathophysiological processes. Studies looking to further characterize the pathophysiology, patient’s demographics and clinical characteristics in the development of strategies for the management of HFpEF and HFmrEF categories are ongoing [21].

Evidence suggests that there is greater therapeutic and mortality benefit derived from higher doses of ACEIs and β-blockers in chronic heart failure (CHF) patients compared to lower doses [22]. Despite this, increasing the titration of HF prognostic therapy to maximally tolerated doses remains poor [23]. A study by Maggioni *et* al. 2015 demonstrated less than a third of HFrEF patients achieved target doses of ACEIs/ARBs and less than 20% received cardio-selective β-blockers therapy. In addition, one-third of patients lacked recorded documentation with regards to reasons for a lack of up titration of medical therapy [24]. Regardless of HF type, there are difficulties in achieving maximal tolerated doses. These gaps have persisted despite HF nurse-led outpatient clinics [25].

Several approaches, including pharmacist-assisted multidisciplinary clinics, have been explored. In previous studies, pharmacist-assisted multidisciplinary management of CHF resulted in significant increase in prescription of GDMT [26], significant reductions in 30- and 90- day all-cause readmissions and HF hospitalizations [27, 28]. This study aimed to evaluate the influence of a pharmacist on prescribing practices of GDMT in CHF patients in a large tertiary hospital over a period of 12 years.

## Methods

This study followed the Strengthening of Reporting of Observational Studies in Epidemiology (STROBE) guidelines [29].

### Study design

This was a retrospective observational study of CHF patients with HFrEF, HFmrEF and HFpEF from two multidisciplinary outpatient clinics in a tertiary referral hospital. These clinics were a Multidisciplinary Ambulatory Consulting Service (MACS) clinic which used a pharmacist-involved model of multidisciplinary care, and a General Cardiology Heart Failure Service (GCHFS) clinic which did not have the active involvement of a pharmacist.

### Setting

This study was conducted at a tertiary metropolitan public hospital in Adelaide, Australia. Secondary data of CHF patients from March 2005 until January 2017 for the MACS clinic patients, and from March 2006 until January 2017 for the GCHFS clinic patients, were collected for this study. There were two systems for the collection and storage of patients’ data within the hospital: MATRIX and OACIS, respectively. MATRIX is a tailored Structured Query Language that allows documentation of comorbidities, medications, patient assessments, and summary of important diagnostic results data management. It allows clinicians to document clinically relevant information, generate evidence-based goals, and to generate letters to patient’s primary care physicians. OACIS (Telus Health, Montreal, Canada) was used as the Patient Administration System for administration of inpatient and outpatient visits, as well as for access to radiology and pathology results.

The in-depth model of care of the MACS clinic is in accordance with a previous publication [30]. The model briefly constitutes a general nursing assessment including blood pressure and weight measurement, pharmacy medication review—followed by a physician review. Physicians involved in the delivery of MACS clinics included Cardiologists, Clinical Pharmacologists, General Physicians, and Geriatricians. Patients managed through the GCHFS were seen by a heart failure-trained nurse and a cardiologist. Both groups of patients had access to a clinical psychologist and an exercise physiologist.

### Participants

Patients primarily diagnosed with HF attending either the MACS clinic or the GCHFS clinic were included. All included patients had previous cardiac imaging supporting a clinical diagnosis of HF. Cardiac imaging modality was predominantly echocardiography although nuclear imaging and cardiac magnetic resonance imaging, along with case notes from external investigations, were also utilized. If the left ventricular function was defined as mildly or more impaired at any time, then patients were classified as having HFrEF. If patients had multiple echocardiography, or other forms of imaging, results demonstrating more severe left ventricular dysfunction were included. Patients were excluded if they did not attend clinic appointments or had incomplete data sets. The overall median follow-up for the study was 1162 days or 3.2 years.

### Variables and outcomes

Outcome variables included patient demographics, clinical characteristics, comorbidities, and prescription practices of GDMT in CHF patients between two clinics. These outcome variables were compared between MACS and GCHFS clinics and across the HFrEF, HFmrEF and HFpEF categories (demographics and clinical characteristics). The age, weight, systolic blood pressure (SBP), diastolic blood pressure (DBP), heart rate (HR), number of medications used, serum creatinine, hemoglobin, mean cell volume (MCV), and comorbidities were measured per patient. The SBP, DBP and HR are the four consecutive readings at rest, five minutes apart, and the average of the last three readings. The data utilized were from the last clinic appointment. The hemoglobin, MCV and creatinine were the last conducted values before first presentation to clinic (which would usually represent the last values before hospital discharge) and the weight was measured at first appointment.

### Outcome measurements

The LVEF value of < 40% for HFrEF, 40–49% for HFmrEF, and ≥ 50% for HFpEF [8] was considered for comparison of demographic, clinical characteristics and comorbidities whereas LVEF value < 40% for HFrEF and ≥ 40% for HFpEF was considered for the evaluation of GDMT. The evaluation of GDMT between HFrEF and HFpEF is clinically significant as there were no separate guidelines for HFmrEF patients in the hospital where this study was conducted for the study duration. To assist evaluation of GDMT, study guidelines were developed based on Australian and European CHF management guidelines (see Additional file 1). Patient data was reviewed for the type of medications prescribed, doses used, and contraindications due to patient characteristics.

Review of all data was performed by two independent investigators (DRP and JEF) to assess for discrepancies as per the developed guidelines (Refer to supplementary file). Differences were resolved by consensus with a third investigator (SS). It was decided that we could not determine the use of GDMT in CHF patients if they did not visit the MACS clinics at least twice. Therefore, for the comparison of the use of GDMT, only patients who had ≥ 2 visit in MACS clinics were included. Polypharmacy was categorized into three groups: non-polypharmacy (0–4 drugs), polypharmacy (5–9 drugs) and hyper-polypharmacy (≥ 10 drugs) as defined by Onder and colleagues [31].

### Study size

**Fig. 1:**
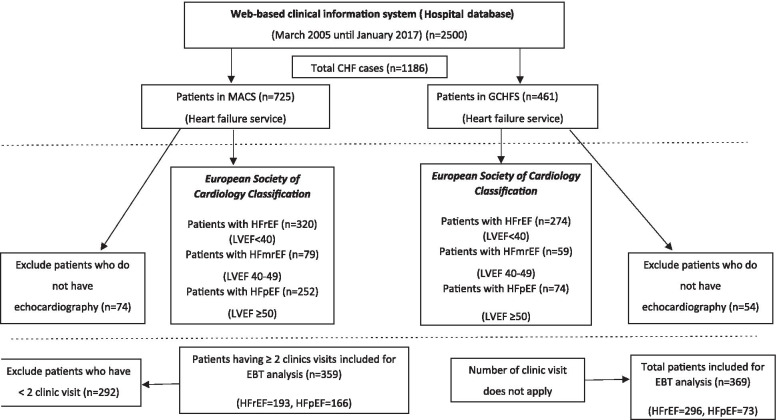
Flow chart of the study. HF, Heart Failure; MACS, Multidisciplinary Ambulatory Consulting Service; GCHFS, General Cardiology Heart Failure Service; LVEF, left ventricular ejection fraction; EBT, evidence-based therapies; HFrEF, heart failure with reduced ejection fraction; HFmrEF, heart failure with mid-range ejection fraction; HFrEF, heart failure with reduced ejection fraction; EBT, evidence-based therapies

During the study period, there were a total of 1186 CHF patients who attended the outpatient clinics and met our eligibility criteria. For the evaluation of GDMT, an individual data of 359 patients from MACS clinic, and 369 patients from the GCHFS clinic were available. Figure 1 details the study flow chart related to the selection of eligible participants.

### Statistical analysis

Statistical analysis was performed using IBM SPSS Statistics for Windows (Version 25.0.0.1. Armonk, NY: IBM Corp). Results are presented as frequency and percentages for categorical variables and median (IQR) for continuous variables. The normal distribution of the numeric variables was assessed using the Shapiro–Wilk test (*p* > 0.05). Median differences between two clinics was evaluated using the Mann–Whitney U test for comparison of demographic and clinical characteristics and use of GDMT between the two clinics—whereas a Kruskal–Wallis test was used for similar comparison among ejection fraction groups—as the data were not normally distributed. Univariate and multivariate binary logistic regression was performed to determine the predictors of evidence-based practice. Nagelkerke R^2^ was used to establish the amount of variance explained by the model. Univariate binary logistic regression was performed to determine the necessary variables to be included in the multivariate analysis. Independent variables which showed a value of < 0.25 in univariate analysis were included in the multivariate analysis. Probability values of *p* < 0.05 were chosen to indicate a statistically significant difference.

## Results

### Participants

A total of 1186 patients were included: 725 patients in the MACS clinic and 461 patients in the GCHFS clinic. 74 patients in the MACS clinic and 54 from the GCHFS clinic who did not have echocardiography were excluded: leaving 651 patients in the MACS clinic and 407 patients in the GCHFS clinic eligible for inclusion (Fig. 1). Two patients from the MACS clinic and thirty-eight patients from the GCHFS clinic were excluded due to incomplete data sets. For the evaluation of GDMT, individual data for 489 HFrEF patients and 239 of the HFpEF patients were reviewed for the type of medications prescribed, doses used, and contraindications due to patient characteristics. The flow diagram of the study is illustrated in Fig. 1.

### Descriptive data

#### Comparison of the differences in demographics, clinical characteristics, and comorbidities by ejection fractions

**Fig. 2:**
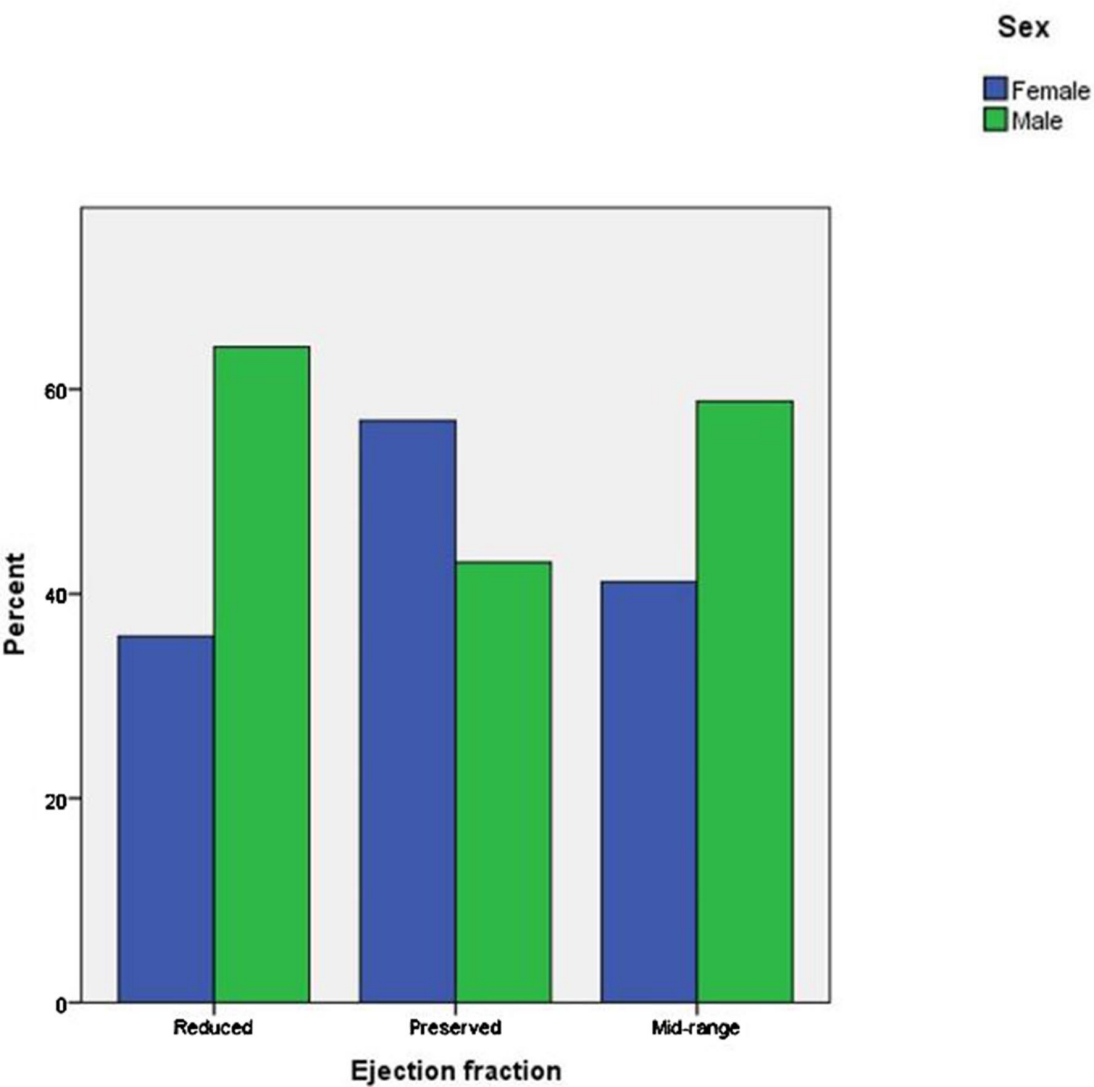
Sex distribution in heart failure with reduced, mid-range and preserved ejection fractions. Kruskal–Wallis test showed a significant difference of sex distribution among three ejection fractions (*p* < .001). *p* < .05 was considered significant

**Fig. 3:**
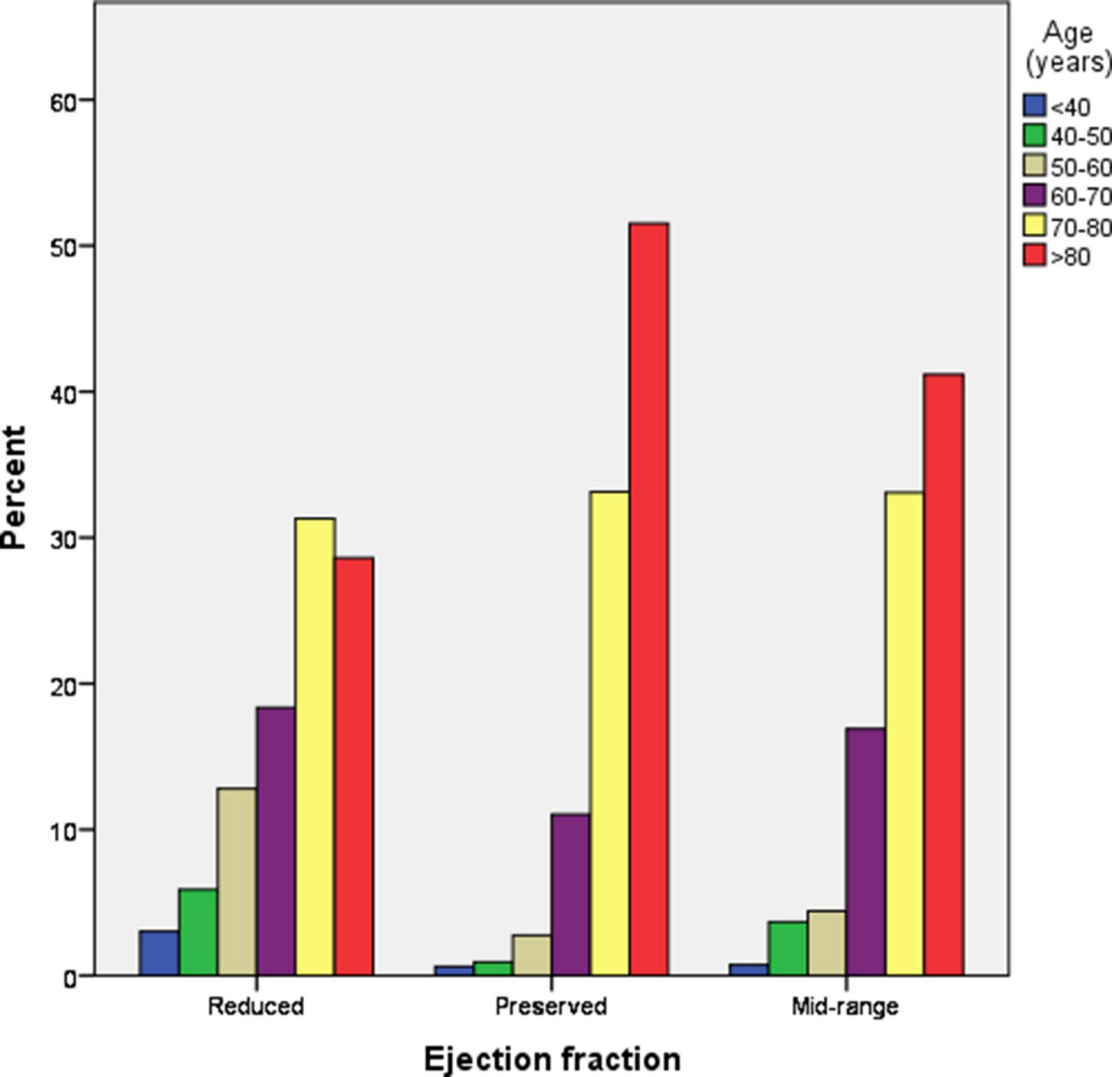
Age distribution in heart failure with reduced, mid-range and preserved ejection fractions. Kruskal–Wallis test showed a significant difference of age distribution among three ejection fractions (*p* < .001). *p* < .05 was considered significant

**Fig. 4:**
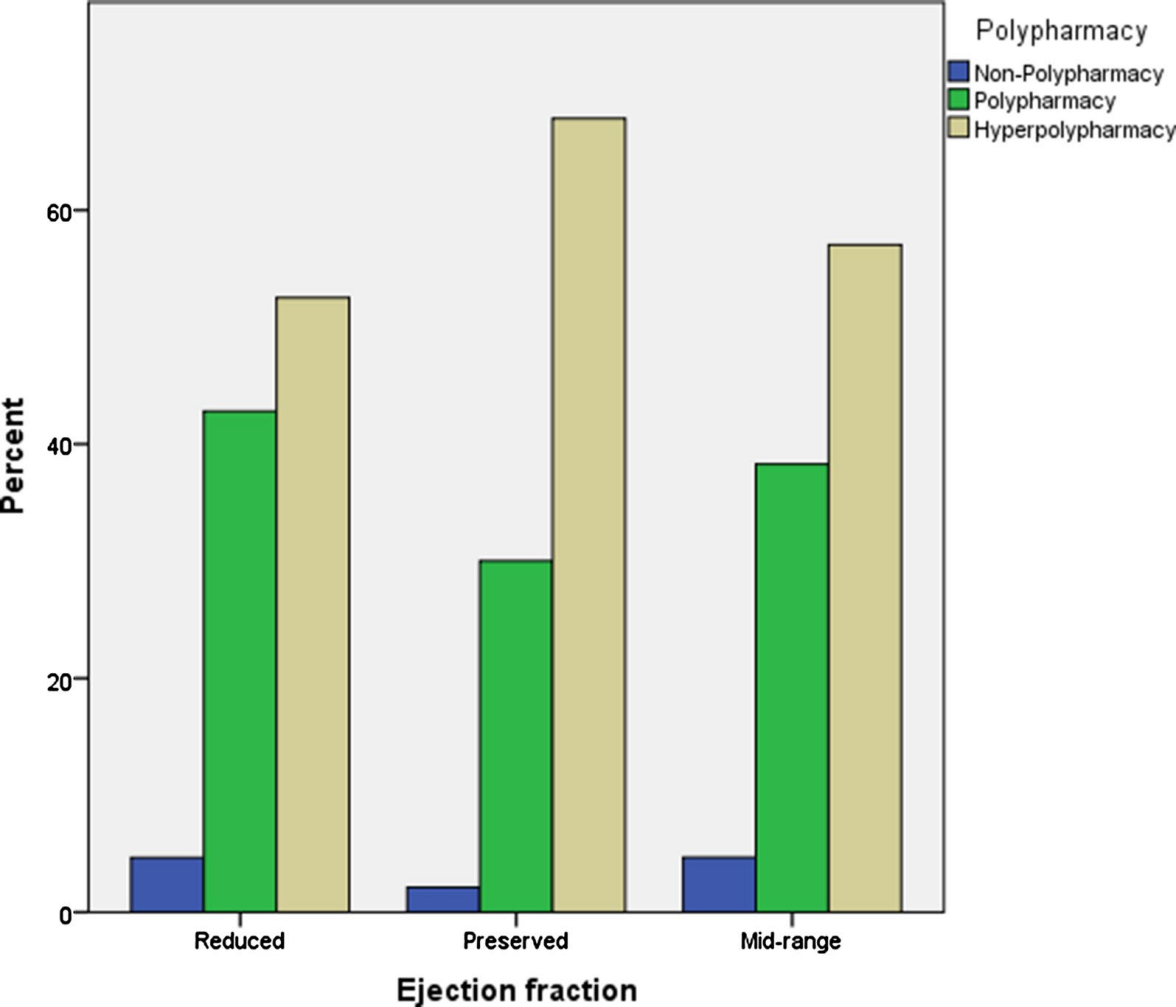
Polypharmacy distribution in heart failure with reduced, mid-range and preserved ejection fractions. Kruskal–Wallis test showed a significant difference of polypharmacy distribution among three ejection fractions (*p* < .001). *p* < .05 was considered significant

Comparison of the demographics and clinical characteristics among the CHF patients stratified by ejection fraction is illustrated in Table 1 and Figs. 2, 3, and 4. The prevalence of HFrEF, HFmrEF and HFpEF was 56%, 13% and 31% respectively (*p* < 0.001) (Table 1). The median age of patients in HFpEF and HFmrEF was significantly greater (*p* < 0.001) than that of the HFrEF cohort. There was no significant difference in the distribution of weight, DBP and HR, serum creatinine and MCV, among HFpEF, HFmrEF and HFrEF group of patients (Table 1). In contrast, a statistically significant difference (*p* < 0.001) was observed for median SBP and number of medications used among HFpEF, HFmrEF and HFrEF group of patients. Hemoglobin levels were highest for HFrEF followed by HFmrEF and HFpEF group. The prevalence of hypertension, AF, osteoarthritis, anemia, and asthma for HFmrEF patients lies between HFrEF and HFpEF, with a higher prevalence of IHD in HFmrEF followed by HFrEF and HFpEF patients (*p* < 0.001) (Table 1). The prevalence of other comorbidities was similar between HFrEF, HFmrEF and HFpEF patients.

**Table 1:** Comparison of demographics and clinical characteristics between ejection fractions

Comorbidities (%)	Total (n = 1056)	Reduced (LVEF < 40) (n = 594)	Mid-range (LVEF = 41–49) (n = 136)	Preserved (LVEF > 50) (n = 326)	*P*-value
Age (years), median (IQR)	78 (68–84)	74 (63–82)	80 (70–84)	81 (75–85.2)	< 0.001***
Weight (Kg), median (IQR)	77 (65–91.2)	76 (65–91)	76.5 (63–93)	80 (66–94)	0.410
SBP (mmHg), median (IQR)	120 (106–135)	114 (101–130)	122 (110–135)	130 (115–144)	< 0.001***
DBP (mmHg), median (IQR)	65 (60–75)	65 (60–76)	65.5 (60–77)	67 (60–75)	0.583
HR (beats/min), median (IQR)	70 (60–80)	71 (61–81)	68 (60–80)	70 (60–81)	0.101
Number of medications used in first appointment, median (IQR)	10 (8–13)	10 (7–13)	10 (8–13)	11 (9–14)	< 0.001***
Serum creatinine (mg/dl), median (IQR)	110 (82–152)	112 (86–154.2)	120.5 (86.2–152)	101 (77.2–142.5)	0.058
Hemoglobin (g/L), median (IQR)	120 (108–134)	124 (111–138)	120 (108–129)	119 (105–131)	0.001**
MCV (fL/red cells), median (IQR)	89.5 (86–93.5)	89.2 (86.2–94)	90.2 (85–94)	89.5 (85–93)	0.736

Comorbidities (%)	Total (n = 1056)	Reduced (LVEF < 40) (n = 594)	Mid-range (LVEF = 41–49) (n = 136)	Preserved (LVEF > 50) (n = 326)	*P*-value
Hypertension	690 (65.3)	329 (55.4)	94 (69.1)	267 (82.0)	< 0.001***
IHD	592 (56.1)	338 (57.0)	87 (64.0)	167 (51.2)	0.035*
AF	497 (47.1)	261 (44.0)	64 (47.1)	172 (53.0)	0.037*
Hyperlipidemia	529 (50.1)	285 (48.0)	79 (58.1)	165 (51.0)	0.102
Diabetes	450 (43.0)	239 (40.2)	58 (43.0)	153 (47.0)	0.145
Osteoarthritis	211 (20.0)	88 (15.0)	29 (21.3)	94 (29.0)	< 0.001***
CRF	328 (3.1)	185 (31.1)	42 (31.0)	101 (31.0)	0.998
COPD	242 (23.0)	127 (21.4)	33 (24.3)	82 (25.2)	0.395
Anemia	204 (19.3)	84 (14.1)	29 (21.3)	91 (28)	< 0.001***
Depression/Anxiety	176 (17.0)	95 (16.0)	19 (14.0)	62 (19)	0.332
Any cardiovascular accident	180 (17.0)	96 (16.2)	25 (18.4)	59 (18.1)	0.685
Asthma	94 (9.0)	41 (7.0)	13 (10.0)	40 (12.3)	0.023*
Any solid caner	155 (15.0)	83 (14.0)	22 (16.2)	50 (15.3)	0.744

Test of significance between reduced, mid-range and preserved ejection fractions groups was performed by Kruskal–Wallis test for all the variables. *p* < .05 was considered significant. LVEF, left ventricular ejection fraction; SBP, systolic blood pressure; DBP, diastolic blood pressure; HR, heart rate; MCV, mean cell volume; IQR, interquartile range; IHD, ischemic heart disease; AF, atrial fibrillation; CRF, chronic renal failure; COPD, chronic obstructive pulmonary disease. *p < .05; **p < .01; ***p < .001

There was a significantly greater number of females diagnosed with CHF in the HFpEF category (Fig. 2). It is noteworthy that half of the patients (51.5%) were > 80 years of age in the HFpEF group followed by HFmrEF (41.2%) and HFrEF (29%) (*p* < 0.001) (Fig. 3). Similarly, the prevalence of hyperpolypharmacy (≥ 10 medications) was highest for the HFpEF group patients followed by HFmrEF and HFrEF patients (Fig. 4).

#### Comparison of the differences in demographics, clinical characteristics, and comorbidities by clinics

Demographic and clinical characteristics of CHF patients compared by clinics are illustrated in Table 2. MACS clinic patients were significantly older (*p* < 0.001), more likely to be female, and had a significantly higher SBP (*p* < 0.001) and DBP (*p* < 0.05) compared to GCHFS clinic patients. Weight and HR were similar between the two clinics. The number of medications used was significantly higher in MACS patients (*p* < 0.001) compared to GCHFS patients. Moreover, the MACS clinic had more polypharmacy and higher prevalence of multiple comorbidities. The prevalence of major comorbidities and proportion of patient with multiple comorbidities were significantly more common in MACS patients compared to GCHFS patients (Table 2).

**Table 2:** Comparison of demographics and clinical characteristics between two clinics

Demographics and clinical characteristics	Total (n = 1184)	MACS (n = 723)	GCHFS (n = 461)	*P*-value
Age (years), median (IQR)	78 (68–84)	80 (72–85)	73 (62–81)	< .001***
Age group (years)				
(< 40)	26 (2.2)	10 (1.4)	16 (3.5)	
(40–50)	49 (4.1)	13 (2.0)	36 (8.0)	
(50–60)	98 (8.3)	41 (6.0)	57 (12.4)	
(60–70)	180 (15.2)	92 (13.0)	88 (19.1)	< .001***
(70–80)	380 (32.1)	242 (33.5)	138 (28)	
(> 80)	451 (38.1)	325 (45.0)	126 (27.3)	
Sex, n (%)				
Male	671 (57.0)	364 (50.3)	307 (67.0)	< .001***
Weight (kg), median (IQR)	77 (65–91.2)	76.34 (63–90)	78 (66–95)	.072
Systolic blood pressure (mmHg), median (IQR)	120 (106–135)	123 (110–140)	112 (100–130)	< .001***
Diastolic blood pressure (mmHg), median (IQR)	65 (60–75)	67 (59–76)	60 (60–71.5)	.010*
Heart rate (beats/min), median (IQR)	70 (60–80)	70 (60–81)	70 (60–80)	.303
Number of medications used, median (IQR)	10 (8–13)	11 (8–14)	9.0 (7.0–12)	< .001***
Non-polypharmacy (0–4 drugs)	44 (4.2)	21 (4.0)	23 (5.0)	
Polypharmacy (5–9 drugs)	406 (39.2)	184 (32.0)	222 (48.3)	< .001***
Hyper polypharmacy (≥ 10 drugs)	586 (57.0)	371 (64.4)	215 (47.0)	
Biochemical parameters				
Serum creatinine (mg/dL), median (IQR)	110 (82–152)	110 (82–151)	124 (97–188)	.305
Hemoglobin (g/L), median (IQR)	120 (108–134)	120 (109–134)	114.5 (104–136)	.538
MCV (fL/red), median (IQR)	89.5 (86–93.5)	89.5 (86–94)	87.6 (82.4–92.5)	.139

Comorbidities	Total (n = 1184)	MACS (n = 723)	GCHFS (n = 461)	*P*-value
Hypertension	769 (65.0)	513 (71.0)	256 (55.5)	< .001***
IHD	660 (56.0)	406 (56.2)	254 (55.1)	.764
AF	549 (46.4)	342 (47.3)	207 (45.0)	.437
Hyperlipidemia	578 (49.0)	350 (48.4)	228 (49.5)	.766
Diabetes	489 (41.3)	328 (45.4)	161 (35.0)	< .001***
Gastroesophageal reflux disease	270 (23.0)	187 (26.0)	83 (18.0)	.002**
Osteoarthritis	231 (19.5)	175 (24.2)	56 (12.1)	< .001***
CRF	359 (30.3)	223 (31.0)	136 (29.5)	.650
COPD	270 (23.0)	199 (27.5)	71 (15.4)	< .001***
Anemia	226 (19.1)	163 (22.5)	63 (14.0)	< .001***
Depression/Anxiety	198 (17.0)	139 (19.2)	59 (13.0)	.004**
Osteoporosis	142 (12.0)	117 (16.2)	25 (5.4)	< .001***
Any cardiovascular accident	197 (17.0)	140 (19.4)	57 (12.4)	.002**
All ophthalmological conditions	124 (10.5)	87 (12.0)	37 (8.0)	.032*
Peripheral vascular disease	160 (13.5)	114 (16.0)	46 (10.0)	.005**
Any solid cancer	176 (15.0)	109 (15.1)	67 (14.5)	.867
Gout	188 (16.0)	125 (17.3)	63 (14.0)	.103
Asthma	108 (9.1)	80 (11.1)	28 (6.1)	.004**
Hypo/Hyperthyroidism	144 (12.2)	102 (14.1)	42 (9.1)	.011*
Thromboembolism	87 (7.3)	65 (9.0)	22 (5.0)	.006 **
Cognitive impairment	87 (7.3)	78 (11.0)	9 (2.0)	< .001***
Proportion of patients with				
≥ 3 comorbidities	1035 (87.4)	665 (92.0)	370 (80.3)	< .001***
≥ 4 comorbidities	895 (76.0)	593 (82.0)	302 (65.5)	< .001***

The median difference was compared using Mann–Whitney U test between MACS and GCHFS groups for age, weight, systolic blood pressure, diastolic blood pressure, heart rate, number of medications, serum creatine, hemoglobin and MCV. Chi-squared test for other categorical variables; age group, sex, polypharmacy, and risk factors was utilized. *p* < .05 was considered significant. MACS, Multidisciplinary Ambulatory Consulting Service; GCHFS, General Cardiology Heart Failure Service; MCV, mean cell volume; IHD, ischemic heart disease; AF, atrial fibrillation; CRF, chronic renal failure; COPD, chronic obstructive pulmonary disease. *p < .05; **p < .01; ***p < .001

#### Comparison of the use of medications in chronic heart failure patients between clinics with heart failure with reduced and preserved ejection fractions

There were similar prescription rates of GDMT between MACS and GCHFS with regards to ACEIs/ARBs in HFrEF patients. It was found that HF patients attending MACS clinic were significantly less likely to be prescribed guideline-directed β-blockers (83.1% vs. 91.1%), MTDs of β-blockers (31.5% vs. 47.3%), and MRAs (32.1% vs. 62.2%)—compared to the GCHFS clinic patients (Table 3). Both clinic patients received similar target doses of ACEIs/ARBs, but MACS clinic patients were less likely to receive target doses of β-blockers compared to those in the GCHFS clinic. Furthermore, the MACS clinic patients had similar rates of prescription for diuretics, but a significantly higher prescription for digoxin in chronic AF (82.5% v. 58.5%) in HFrEF patients.

**Table 3:** Comparison of the use of medications between clinics with heart failure with reduced ejection fraction (EF < 40) patients

Use of medications	Total (n = 489)	MACS (n = 193)	GCHFS (n = 296)	*P*-value
Contraindications for ACEIs	69 (14.1)	44 (23.0)	25 (8.4)	< .001***
Rate of appropriate use of ACEIs	280 (67.0)	97 (65.1)	183 (67.5)	.617
Contraindications for MTD of ACEIs	69 (14.1)	44 (23.0)	25 (8.4)	< .001***
Rate of appropriate use of MTD of ACEIs	193 (46.0)	64 (43.0)	129 (48.0)	.325
Target dose used for ACEIs	175 (36.0)	60 (31.1)	115 (39.0)	.080
Rate of appropriate use of ACEIs/ARBs	297 (71.0)	102 (68.4)	195 (72.0)	.438
Rate of appropriate use of MTD of ACEIs/ARBs	210 (50.0)	69 (46.3)	141 (52.0)	.264
Target dose of ACEIs/ARBs	203 (41.5)	71 (37.0)	132 (45.0)	.157
Contraindications for β-blockers	11 (2.2)	9 (5.0)	2 (1.0)	.006**
Rate of appropriate use of β-blockers	421 (88.1)	153 (83.1)	268 (91.1)	.008**
Contraindications for MTD of β-blockers	11 (2.2)	9 (5.0)	2 (1.0)	.006**
Rate of appropriate use of MTD of β-blockers	197 (41.2)	58 (31.5)	139 (47.3)	< .001***
Target dose used for β-blockers	151 (31.0)	42 (22.0)	109 (37.0)	< .001***
MRA contraindications	34 (7.0)	31 (16.1)	3 (1.0)	< .001***
MRA used without contraindications	246 (50.3)	62 (32.1)	184 (62.2)	< .001***
Diuretics contraindications	2 (0.41)	2 (1.0)	0 (0)	–
Diuretics used without contraindications	419 (86.0)	162 (84.0)	257 (87.0)	.373
Digoxin contraindications	7 (6.0)	2 (1.0)	5 (1.7)	.552
Digoxin use without contraindications	112 (25.0)	57 (30.0)	65 (22.0)	.059
Use of digoxin in chronic atrial fibrillation	85 (70.0)	47 (82.5)	38 (58.5)	.004**

The group difference was evaluated using Chi-square (χ^2^) test. *p* < .05 is considered significant. EF, ejection fraction; MACS, Multidisciplinary Ambulatory Consulting Service; GCHFS, General Cardiology Heart Failure Service; ACEIs, angiotensin-converting enzyme inhibitors; MTDs, maximum tolerated doses; ARBs, angiotensin receptor blockers; MRAs, mineralocorticoid receptor antagonists. **p < .01; ***p < .001

Rate of appropriate use of ACEIs was calculated in percentage as the number of patients who received the ACEIs without any contraindications, divided by the number of patients who should have received the ACEIs. The rate of appropriate MTD use of ACEIs was calculated as the number of patients who received the MTD of ACEIs without any contraindications, divided by the number of patients who should have received the MTD of ACEIs. The maximum tolerated dose was calculated as the dose given as a percentage of the target dose. The rate of appropriate use of β-blockers, MTD of β-blockers and target dose of β-blockers were calculated similarly to that of appropriate use of ACEIs, MTD of ACEIs, and target dose of ACEIs

For patients with HFpEF, a significantly higher prescriptions of ACEIs/ARBs (70.5% vs. 56.2%) in MACS clinic, but a significantly lower prescriptions of β-blockers (54% vs. 68.5%), MRAs (30.1% vs. 48%), furosemide and anticoagulation for AF, were observed in the MACS clinic patients compared to those in the GCHFS clinic (Table 4). However, a similar prescription rate for digoxin was seen between two clinics.

**Table 4:** Comparison of the use of medications between clinics with heart failure with preserved ejection fraction (EF > 40) patients

Use of medications	Total (n = 239)	MACS (n = 166)	GCHFS (n = 73)	*P*-value
ACEIs use	94 (39.3)	71 (43.0)	23 (31.5)	.101
ARB use	64 (27.0)	46 (28.0)	18 (25.0)	.623
ACEIs/ARBs	155 (65.0)	117 (70.5)	41 (56.2)	0.0314*
β-blockers use	139 (58.2)	89 (54.0)	50 (68.5)	.032*
MRA use	85 (35.6)	50 (30.1)	35 (48.0)	.008**
Furosemide	208 (87.0)	139 (84.0)	69 (94.5)	.022*
Digoxin used	60 (25.1)	42 (25.3)	18 (25.0)	.916
Contraindications for anticoagulation in AF	12 (5.0)	10 (6.0)	2 (3.0)	.006**
Anticoagulated in presence of AF without contraindications	80 (33.5)	45 (27.1)	35 (48.0)	.002**

The difference between the clinics was evaluated using Chi-square (χ^2^) test. Pearson Chi-square < .05 is considered significant. EF, ejection fraction; MACS, Multidisciplinary Ambulatory Consulting Service; GCHFS, General Cardiology Heart Failure Service; ACEIs, angiotensin-converting enzyme inhibitors; MTDs, maximum tolerated doses; ARBs, angiotensin receptor antagonists; MRAs, mineralocorticoid receptor blockers; AF, atrial fibrillation. *p < .05; **p < .01

#### Predictors of ACEIs/ARBs, β-blockers, MRAs and maximum tolerated dose of ACEIs/ARBs and β-blockers use in heart failure with reduced ejection fraction (EF < 40) patients

Age, last clinic SBP, last clinic DBP, AF, anaemia, IHD, CRF, COPD, any cognitive impairment, any solid cancer, any CVA, falls, osteoarthritis, GORD, peripheral vascular disease (PVD), gout, ≥ 3 comorbidities and any thyroid disease being the significant predictors in the univariate analysis, were included in multivariate analysis of ACEIs/ARBs use (data not shown). Nagelkerke R^2^ showed that the above variables used in the multivariate binary logistic analysis model could explain 26.4% in predicting the practice of ACEIs/ARBs use. Age, anaemia, CRF, gout and GORD were the negative predictors, whereas SBP was a positive predictor for the use of ACEIs/ARBs in HFrEF patients in the multivariate analysis (Table 5). Similarly, age, AF, IHD, CRF, COPD, any solid cancer, osteoarthritis, GORD, gout and presence of ≥ 3 comorbidities being the significant predictors in the univariate analysis, were included in multivariate analysis of the MTD use of ACEIs/ARBs (data not shown). The model explained 13.4% (Nagelkerke R^2^) in predicting the practice of MTD of ACEIs/ARBs. Age and CRF were significant negative predictors of the use of MTD of ACEIs/ARBs in the multivariate analysis (Table 5).

**Table 5:** Multivariate binary logistic regression for the use of ACEIs/ARBs, β-blockers, and MRAs in heart failure with reduced ejection fraction (EF < 40) patients

Variables	B	Sig	Exp (B)	95% CI for Exp (B)	
				Lower	Upper
ACEIs/ARBs in heart failure with reduced ejection fraction					
Age	− .033	.010	.968	.944	.992
Last clinic SBP	.020	.020	1.020	1.003	1.037
Anemia	− .707	.024	.493	.267	.910
CRF	− 1.228	.000	.293	.174	.492
Gout	− .613	.044	.542	.298	.983
GORD	− .602	.033	.548	.315	.952
MTD of ACEIs/ARBs in heart failure with reduced ejection fraction					
Age	− .032	.000	.968	.952	.984
CRF	− .602	.010	.548	.346	.867
β-blockers in heart failure with reduced ejection fraction					
Last clinic HR	− .028	.007	.973	.953	.992
Gout	− .891	.013	.410	.203	.828
IHD	.583	.048	1.792	1.006	3.191
MTD of β-blockers in heart failure with reduced ejection fraction					
Age	− .040	.000	.961	.943	.979
Last clinic HR	− .026	.003	.974	.958	.991
MRAs use in heart failure with reduced ejection fraction					
Age	− .040	.000	.960	.942	.979
Last clinic SBP	− .034	.000	.966	.955	.978

Variable (s) entered on step 1: last clinic SBP, last clinic DBP, any anemia, CRF, gout, IHD, GORD, any solid cancer, any CVA, PVD, OA, falls, any cognitive impairment, COPD, AF and any thyroid

EF, ejection fraction; SBP, systolic blood pressure; DBP, diastolic blood pressure; CRF, chronic renal failure; IHD, ischemic heart diseases; GORD, gastroesophageal reflux diseases; CVA, cardiovascular accident; PVD, peripheral vascular disease; COPD, chronic obstructive pulmonary diseases; AF, atrial fibrillation; ACEIs/ARBs, angiotensin converting enzyme inhibitor/angiotensin receptor antagonists; MTD, maximum tolerated dose; MRAs, mineralocorticoid receptor blockers; HR, heart rate; COPD, chronic obstructive pulmonary diseases. *p* < .05 is considered significant

Only the significant variables in multivariate analysis are shown

Age, sex, HR, COPD, any solid cancer, gout, any anemia, IHD, any cognitive impairment, osteoporosis and any thyroid diseases being the significant predictors in the univariate analysis, were included in multivariate analysis of β-blocker use (data not shown). The model explained 12.9% (Nagelkerke R^2^) in predicting the use of f β-blockers. HR and gout were significant negative predictors, but IHD was a significant positive predictor for the use of β-blockers in HFrEF patients in the multivariate analysis (Table 5).

Age, sex (male), last clinic SBP, last clinic DBP, last clinic postural BP, AF, anemia, CRF, hypertension, any cognitive impairment, any solid cancer, hyperlipidemia, falls, osteoarthritis, osteoporosis, PVD and ≥ 3 comorbidities, being the significant predictors in the univariate analysis, were included in multivariate analysis of MRA use (data not shown). The model explained 26.4% (Nagelkerke R^2^) in predicting the use of MRA. Age and SBP were the significant negative predictors for the use of MRAs in HFrEF patients in the multivariate analysis (Table 5).

#### Predictors of use of ACEIs/ARBs, β-blockers and MRAs in heart failure with preserved ejection fraction (EF > 40) patients

Sex (male), hypertension, CRF, CVA, COPD, cognitive impairment, gout, and falls were significant predictors (*p* < 0.25) in the univariate analysis, being the significant predictors in the univariate analysis, were included in the multivariate analysis of ACEIs/ARBs (data not shown). The model explained 18.5% (Nagelkerke R^2^) in predicting the use of ACEIs/ARBs. CRF, and cognitive impairment were the significant negative predictors, but hypertension and COPD were the significant positive predictor for the use of ACEIs/ARBs in the multivariate analysis of the HFpEF patients (Table 5).

Hypertension, last clinic HR, last clinic low heart rate (HR < 60), anemia, IHD, diabetes, COPD, cognitive impairment, hyperlipidemia, osteoarthritis and GORD, being the significant predictors in the univariate analysis, were included in multivariate analysis of β-blockers use (data not shown). The model explained 30.1% (Nagelkerke R^2^) in predicting the use of β-blockers. HR, COPD and GORD were the significant negative predictors, but IHD was the significant positive predictor for the use of β-blockers in the multivariate analysis of the HFpEF patients (Table 6).

**Table 6:** Multivariate binary logistic regression for the use of ACEIs/ARBs, β-blockers, and MRAs in heart failure with preserved ejection fraction (EF > 40) patients

Variables	B	Sig	Exp (B)	95% C.I. for EXP (B)	
				Lower	Upper
ACEIs/ARBs in heart failure with preserved ejection fraction					
Hypertension	1.238	.001	3.449	1.677	7.095
CRF	− .686	.036	.504	.266	.955
COPD	.871	.023	2.389	1.129	5.055
Any cognitive impairment	− 1.509	.012	.221	.068	.719
β-blockers in heart failure with preserved ejection fraction					
Last clinic HR	− .040	.009	.961	.933	.990
IHD	.740	.023	2.096	1.106	3.971
COPD	− 1.262	.001	.283	.137	.584
GORD	− .681	.043	.506	.262	.980
MRAs in heart failure with preserved ejection fraction					
Low standing SBP (BP < 115)	.744	.037	2.105	1.044	4.244

Variable (s) entered on step 1: CRF, any CVA, COPD, any cognitive impairment, gout, and falls. EF, ejection fraction; CRF, chronic renal failure; CVA, cardiovascular accident; COPD, chronic obstructive pulmonary diseases; ACEIs/ARBs, angiotensin converting enzyme inhibitor/angiotensin receptor antagonists. ACEIs/ARBs, angiotensin converting enzyme inhibitor/angiotensin receptor antagonists; MTD, maximum tolerated dose; MRAs, mineralocorticoid receptor blockers. *p* < .05 is considered significant

Only the significant variables in multivariate analysis are shown

Sex, hypertension, AF, IHD, diabetes, CRF, asthma, hyperlipidemia, osteoporosis, and low standing SBP, being the significant predictors in the univariate analysis, were included in multivariate analysis of MRAs use in HFpEF patients (data not shown). The model explained 15.5% (Nagelkerke R^2^) in predicting the use of MRAs. Only the low standing SBP was a significant positive predictor for the use of MRAs in the multivariate analysis (Table 6).

## Discussion

This study is a detailed analysis of demographics, clinical characteristics, comorbidities, and prescribing practice of GDMT in CHF outpatients in a large tertiary metropolitan hospital in South Australia. The HFmrEF subjects resembled the HFpEF patients in terms of age, HR, SBP and having higher prevalence of polypharmacy, as well as resembled the HFrEF cohort for the proportion of male distribution and prevalence of IHD. Contrary to our hypothesis, the pharmacist-involved multidisciplinary team had significantly lower rates of prescription of β-blockers, MTD of β-blockers, a target dose of β-blockers, and MRAs prescribed in the both the HFrEF and HFpEF patients as compared to the GCHFS clinic. Clinically important predictors of lower utilization of evidence-based medication in MACS clinic are further described below.

The HFrEF, HFmrEF and HFpEF patients in this study were notably older than the ESC Heart Failure Long-Term (ESC-HF-LT) registry [32]. HFpEF patients in the current study were 2 years older but one year younger for the HFrEF group compared with the age of patients in the NSW (Australia) snapshot study [33]. AF prevalence in the current study was in ascending order with the increasing value of LVEF, as also found in the Swedish Heart Failure Registry [34]. In line with our results, similar findings to the HFmrEF group resembling HFrEF for male sex, and IHD, were reported in earlier studies [35, 36]. Anemia is also a contributory factor to symptoms in HFpEF [37], so it is not surprising that hemoglobin levels were lower and the incidence of anemia higher in this group. Difference in IHD is likely by chance alone. Differences in SBP between clinics likely reflects different ratios of HFrEF vs HFpEF seen in each clinic, as well as the use of medications. This current study found a notable difference in demographics and comorbidities with the different cut-offs for EF. Based on above-mentioned results, this study showed intermediate demographic and clinical characteristics for HFmrEF category between HFpEF and HFrEF. Other studies showed that digoxin reduces HF hospitalization [38, 39], but does not have any mortality benefits. As a result, digoxin has fallen out of favor in the management of HFrEF, although there appears a role for rate-control of AF in patients who may not have a high baseline blood pressure e.g. patients with HFrEF. This could be a potential reason why this study observed a higher prevalence of digoxin use in the MACS clinic patients (HFrEF) than in the GCHFS clinic.

GDMT use was higher in the current study compared to the NSW HF snapshot study for the use of ACEIs/ARBs, β-blockers and MRAs in HFrEF patients [33]. Similar patterns of better use of ACEIs/ARBs, β-blockers and MRAs were evident, but there were slightly lower rates of prescription of diuretics and digoxin observed in this study compared to another Australian study on chronic HFrEF patients [40]. Importantly, the prescription of ACEIs/ARBs, β-blockers and MRAs were similar or even superior to previous studies conducted in Australia in HFrEF patients [33, 40]. However, higher prescription rate for ACEIs/ARBs and β-blockers have been reported in studies conducted in the USA [41] and Europe [24]—than in our findings.

MACS being a multidisciplinary service, combines evidence-based clinical guidelines, patient centred practice, and a shared care approach. The MACS clinic is less likely to prescribe ACEI/s/ARBs due to a variety of reasons as demonstrated by previous studies such as old age [42], risk of anaemia [43], worsening of renal function [44], contraindications for their use [24], adverse effects associated with higher doses compared to lower doses for ACEIs/ARBs [45], and concomitant use of other mediations due to presence of multiple comorbidities [46]. We hypothesized that the MACS clinic has higher prescription of ACEIs/ARBs than in the GCHFS clinic. However, older age, anaemia and CRF being the significant negative predictors, both the clinic patients received similar rates of prescription. Additionally, a greater number of contraindications for the use of ACEIs/ARBs and presence of polypharmacy were important factors to be considered in the MACS clinic compared to GCHFS clinic. Notably, gout and GORD are negative significant predictors for the utilization of ACEIs/ARBs in HFrEF patients which have not been reported before in the literature. Even though SBP was a significant positive predictor, and MACS clinic patients had a higher SBP, the impact of negative predictors and other variables as explained above was superior for the utilization of ACEIs/ARBs. Age and presence of CRF were significant negative predictors for the MTD use of ACEIs/ARBs.

Compelling evidence exists regarding underutilization of β-blockers and failure to up-titration in CHF patients including older age (> 70 years) and presence of respiratory disease [47], hypotension and polypharmacy [48], concern of side effects, contraindications, poor experience of GPs [49], and low HR and poor adherence to prescriptions [50]. Patients receiving β-blockers may experience adverse effects-mortality and cardiovascular events associated with high resting HR—as described by Chen and colleagues [51]. Nevertheless, the reluctance of the clinicians to prescribe β-blockers due to potential side effects requires further investigation. Consistent with previous findings, HR was a significant negative predictor for the use of β-blocker and HR and older age were the negative significant predictors for the MTD use of β-blockers. Other potential reasons for the lower utilization of β-blockers, despite the presence of a pharmacist in the MACS clinic, could be a higher prevalence of polypharmacy due to existing comorbidities [48] and the presence of contraindication for their use [24, 49]. Patients were significantly older, and the prevalence of gout were significantly higher, but IHD and HR were similar in HFrEF patients between the two clinics. Notably, gout as a significant negative predictor and GORD as a significant positive predictor for the utilization of β-blocker, were found in HFrEF patients. This finding has not been reported before in the literature. In certain instances, the underlying reason for the underutilisation of GDMT may also be unknown.

A major reason behind the underutilisation of MRAs in HF is due to associated hyperkalaemia and the detrimental effect on renal function as reported earlier [52]. In contrast, renal function was not a significant predictor in our findings. However, patient age and last clinic SBP were significant negative predictors for the utilisation of MRAs. There were more patients experiencing contraindications in the use of MRAs in the MACS clinic than the GCHFS clinic patients, which appeared to impact lower prescription. Further research is needed to confirm other relevant reasons, for example, the occurrence of hyperkalaemia. Presence of digoxin use is more likely if patients have AF [40] as it improves morbidity in HF patients [53]. One key advantage of a pharmacist being at the MACS clinic was that a significantly higher number of patients received digoxin than in the GCHFS clinic in HFrEF patients.

Our study found that 41.5% of patients were given the recommended target dose for ACEIs/ARBs and 31% of patients received recommended doses of β-blockers. Overall, it is important to note that the rate of target dose prescribed was superior to larger studies conducted in Europe [22, 24], and in Asia [54]. It is recommended that the tolerability of specific doses in individual patients with multiple comorbidities and polypharmacy, in HF patients, should be closely monitored rather than just an approach to reach the target doses [55, 56]. It is crucial that the emphasis for up-titration should be adopted based on an individualised dose approach. According to one systematic review, the widely recognised definition of polypharmacy is a condition that requires the use of five or more medications daily (range = 2 to 11) [57]. The mean number of medications used in the MACS patients was 11 ± 4, which is much higher than reported indicating that there was substantial polypharmacy in MACS clinic patients. Lower GDMT use in HFrEF patients due to underlying contraindications have been reported previously [58]. The contraindications for use of ACEIs, MTDs of ACEIs, β-blockers and MTD of β-blockers were significantly higher in MACS clinic patients than in GCHFS clinic patients. It can be argued that where many patients have contraindications, clinicians are more reluctant to prescribe GDMT due to lack of more extensive experience of appropriate dosing than when patients do not have contraindications. Such findings highlight that contraindications may be one potential reason for lower utilization of GDMT, in the MACS clinic, despite the pharmacist’s active involvement.

Despite caveats relating to difficulties in understanding the epidemiology, pathophysiology and paucity of evidence for the effective management of HFpEF, expert groups have highlighted that the successful management of HFpEF has been partly addressed due to the possible benefits of currently available medications [59]. In contrast to HFrEF patients, a significantly higher prescription of ACEIs/ARBs but significantly lower prescription of β-blockers and MRAs, in the MACS clinic patients compared to GCHFS clinic, was observed in HFpEF patients. The higher prescription of ACEI/ARBs may be due to underlying left atrial hypertension and pulmonary hypertension as explained by Lam and colleagues [60], given the higher number of HFpEF patients in the MACS clinic. Hypertension and COPD are the significant positive predictors whereas CRF and cognitive impairment were the significant negative predictors for the utilization of ACEIs/ARBs in this study. Consistent to the current study, a previous Australian study demonstrated a significantly lower prescription of ACEIs in HFpEF patients compared to those in HFrEF patients [61]. These findings indicate that patients in the HFpEF category in this study were not over-treated. It has also been reported that age is a strong predictor of the lower prescription of β-blockers in the elderly in HFpEF patients [62]. The presence of COPD, gout and last clinic HR were significant positive predictors for the lower use of β-blockers in HFpEF. However, the presence of IHD was a significant positive predictor for the use of β-blockers. Again, the differential prevalence of these comorbidities between the MACS and GCHFS clinics explains why MACS patients have significantly lower prescriptions of β-blockers in this study. The low standing SBP was associated with a higher prescription of MRAs in HFpEF patients. Indeed, effectiveness of currently available GDMT for HFpEF is still controversial.

A systematic review and meta-analysis revealed that the use of MRAs in HFpEF was associated with ADRs including hyperkalaemia and gynecomastia compared with HFrEF patients [63]. The exact benefits of MRAs in HFpEF patients, however, is still poorly understood [64]; therefore, the generalisation of the role of currently available medications may not be clinically relevant. The MACS clinic, being a holistic model of care, may have considered these ADRs in prescribing MRAs in HFpEF patients, which could be a potential reason for a significantly lower prescription of MRAs in the MACS clinic compared with in the GCHFS clinic. Some cases of inappropriate prescribing were identified in the GCHFS clinic; for example, two patients were prescribed two β-blockers simultaneously, while two other patients were on both ACEIs and ARBs and one patient received the wrong dose of apixaban. Similarly, some patients were on contraindicated medications. The benefit of having a pharmacist in the multidisciplinary team is that pharmacists are more likely to detect cases of inappropriate prescribing and more accurately identify contraindicated medications.

A previous study using the data of MACS clinic showed that clinicians were adhering to clinical guidelines despite a higher number of patients with multiple comorbidities [65]. Additionally, another study using the same clinic data reported that, despite patients having multiple comorbidities, there was improved survivability [30]. Based on previous findings, it is likely that MACS clinics do have a survival benefit but requires further investigation. Such a proposed study is currently seeking funding. Demographic and clinical characteristics, contraindications for their use, polypharmacy, and underlying comorbidities determines the best practice approach of evidence-based medications in CHF patients.

### Strengths and limitations

This study encompasses a large number of patients over 12 years from a large tertiary hospital. All patients included had cardiac imaging confirming left ventricular function. The most important confounder in this study was the series of different guidelines for the management of CHF across the study duration. Additionally, there was a large range of expertise levels, practice behaviours, practice duration of clinicians, nursing staff and pharmacists in this study. Evaluation of these potential confounders was beyond the scope of this study. The main bias here was the referral bias, where different types of patients may be referred to the two different clinics. As there were no separate guidelines for HFmrEF patients in the hospital where this study was conducted, evaluation of GDMT was only considered for HFrEF and HFpEF in this analysis. Due to the limitation of funding and study completion timeline, differences in hospitalizations and survivability were not evaluated—although they are identified as important clinical outcomes.

## Conclusions

The older age of patients, heart rate, blood pressures, renal dysfunctions, contraindications for use of GDMT, and polypharmacy were the main potential reasons for lower prescription of β-blockers and MRAs in the MACS clinic in both HFrEF and HFpEF patients. The other roles of the pharmacist within a multidisciplinary team, including continuity of care, medication compliance, prevention of adverse reactions, and non-pharmacological compliance require further investigation.

## Supplementary Information

**Additional file 1.** Guidelines for analysis of Guideline-directed medical therapy (GDMT) for heart failure with reduced and preserved ejection fraction.

## Data Availability

The datasets used and/or analyzed during the current study are available from the corresponding author on reasonable request.

## Abbreviations

GDMT: Guideline-directed medical therapy
HF: Heart failure
CHF: Chronic heart failure
MACS: Multidisciplinary Ambulatory Consulting Service
GCHFS: General Cardiology Heart Failure Service
HFmrEF: Heart failure with mid-range ejection fraction
HFpEF: Heart failure with preserved ejection fraction
HFrEF: Heart failure with reduced ejection fraction
HR: Heart rate
SBP: Systolic blood pressure
DBP: Diastolic blood pressure
MCV: Mean cell volume
IHD: Ischemic heart disease
ACEIs: Angiotensin-converting enzyme inhibitors
ARB: Angiotensin receptor blockers
MRAs: Mineralocorticoid receptor antagonists
AF: Atrial fibrillation
LVEF: Left ventricular ejection fraction (LVEF)
STROBE: Strengthening the Reporting of Observational Studies in Epidemiology
