# Supplementary material for: Evaluation of the prescribing practice of guideline-directed medical therapy among ambulatory chronic heart failure patients
Source: BMC Cardiovasc Disord. 2021 Feb 18;21:104. doi: 10.1186/s12872-021-01868-z (PMC7893887; doi:10.1186/s12872-021-01868-z)
Supplement: Supplementary file 1 — Additional file 1. Guidelines for analysis of Guideline-directed medical therapy (GDMT) for heart failure with reduced and preserved ejection fraction. [file 12872_2021_1868_MOESM1_ESM.docx]

**Additional file 1: Guidelines for analysis of Guideline-directed medical therapy (GDMT) for heart failure with reduced and preserved ejection fraction**

These criteria were developed based on 2016 European Society of Cardiology guidelines for indications, and contraindications. However, the dose of each individual drugs was based on Australian Medicine Handbook. Review of all data was performed by two independent investigators (DRP and JEF) to assess for discrepancies as per the developed guidelines. Differences were resolved by consensus with a third investigator (SS). Standard criteria for evaluation of use of different medications in HFrEF and HFpEF are described below.

**A. Standard criteria for heart failure with reduced ejection fraction (HFrEF)**

**Angiotensin converting enzyme inhibitors (ACEIs) inhibitors use or not**

**3**= where there is no indication for ACEIs, or there is contraindication to its use

**1**= where there is indication and any ACEIs dose is prescribed

**0**= where there is indication and no contraindication and ACEIs are not prescribed

**2=** where there is use of ACEIs in presence of clear contraindication such as angioedema, pregnancy, intolerance, or hypersensitivity to ACEIs

**Indication for ACEIs** is EF<40%

**Contraindications to ACEIs inhibitors are:**

- *Bilateral renal artery stenosis
- Angioedema
- Pregnancy
- Presence of intolerance or hypersensitivity to ACEIs
- *Significant renal dysfunction; serum creatinine >221µ mol/L or 2.5 mg/dL or eGFR<30 mL/min/1.73m^2^
- *Systolic BP on sitting or standing of <90 (whichever is less)

*=Does not apply to patients already on ACEIs

**ACEIs dose maximum tolerated dose (MTD)** (only for those who are on ACEIs)

**3**= where there is no indication for maximum tolerated dose of ACEIs, or there is contraindication for maximum tolerated dose.

**1=** where there is indication, and maximum tolerated dose of ACEIs are prescribed

**0**= where there is indication, no contraindication and maximum tolerated dose of ACEIs are not prescribed.

**2=** where there is use of MTD of ACEIs in presence of clear contraindication such as angioedema, pregnancy, intolerance, or hypersensitivity to ACEIs.

**% of maximum tolerated dose who are on ACEIs**

- Calculated as the dose given as percentage of lower target dose
- Should be calculated for all cases where ACEIs or not is not blank
- Although the daily dose used is greater than target dose, the maximum % of MTD is 100%

**Starting dose and Target dose for Angiotensin converting enzyme inhibitors (ACEIs) inhibitors**

|  | Starting dose (mg) | Target dose (mg) |
| --- | --- | --- |
| ACEIs doses | | |
| Captopril | 6.25 t.i.d. | 50 t.i.d. |
| Enalapril | 2.5 o.d. | 10-20 o. d. |
| Lisonopril | 2.5 o.d. | 20-40 o.d. |
| Ramipril | 2.5 b.d. | 10 o.d. |
| Trandolapril | 1 o.d. | 4 o.d. |
| Perindopril arginine salt | 2.5 o.d. | 5 o.d. |
| Perindopril erbumin salt | 2 o.d. | 4 o.d. |
| Fosinopril | 5-10 o.d. | 10-40 o.d. |

**β –blockers use or not**

**2**= where there is no indication for β –blockers, or there is contraindication to its use

**1**= where there is indication and any β –blockers dose is prescribed

**0**= where there is indication and no contraindication and β –blockers are not prescribed

**Indication for β –blockers** is EF<40%

**Contraindications to β –blockers are:**

- Second- or third-degree AV block (in the absence of a pacemaker).
- Critical limb ischemia
- Severe or poorly controlled asthma
- Known allergic reactions/other adverse reaction (drug specific)
- *severe hypotension; systemic blood pressure <90mm Hg (lower of sitting or standing)
- *Heart rate <50 beats/minute (bpm unless a pacemaker is present).

*=Does not apply to patients already on β –blockers

**Whether β –blockers dose maximum tolerated dose (MTD)**

**2**= where there is no indication for maximum tolerated dose of β –blockers, or there is contraindication for maximum tolerated dose

**1=** where there is indication, and maximum tolerated dose of β –blockers are prescribed

**0**= where there is indication, no contraindication and maximum tolerated dose of β –blockers are not prescribed.

**% of maximum tolerated β –blockers dose**

- Calculated as the dose given as percentage of lower target dose
- Should be calculated for all cases where β –blockers or not is not blank
- If actual daily dose is >100% of target dose, then only put in 100%

**Starting dose and target dose of β –blockers**

|  | Starting dose (mg) | Target dose (mg) |
| --- | --- | --- |
| β –blocker | | |
| Bisoprolol | 1.25 o.d. | 10 o.d. |
| Carvedilol | 3.125 b.i.d. | 25 b.i.d. |
| Metoprolol succinate (CR/XL) | 23.75 o.d. | 190 o.d. |
| Metoprolol | 12.5 b.d. | 100 b.d. |
| Nebivolol | 1.25 o.d. | 10 o.d. |
| Atenolol | 20 o.d. | 100 o.d. |

**Angiotensin receptor blockers (ARBs) use or not**

**1=** ARB used

**0=**ARB not used

**ARBs EBM use or not**

**3**= where there is no indication for ARBs, or there is contraindication to its use

**1**= where there is indication, no contraindication and any ARBs dose is prescribed

**0**= where there is indication, no contraindication and ARBs are not prescribed

**2=** where the patient is on an ARB but really there is no contraindication to ACEIs, and they should be on that instead

**Indication for ARBs** is EF<40%, and there is documented intolerance to ACEIs

**Contraindications to ARBs inhibitors:**

- Pregnancy
- Bilateral renal artery stenosis
- previous intolerance or hypersensitivity to ARBs
- Significant renal dysfunction; serum creatinine >221µ mol/L or 2.5 mg/dL or eGFR<30 mL/min/1.73m^2^
- Systolic BP on sitting or standing of <90 (whichever is less)

**Whether ARBs dose maximum tolerated dose (MTD)**

**3**= where there is no indication for maximum tolerated dose of ARBs, or there is contraindication for maximum tolerated dose

**1=** where there is indication and maximum tolerated dose of ARBs are prescribed

**0**= where there is indication and no contraindication and maximum tolerated dose of ARBs are not prescribed.

**2=** where the patient is on an ARB at maximum tolerated dose without documented intolerance to ACEIs

**% of maximum tolerated ARBs dose**

• Calculated as the dose given as percentage of lower target dose

• Should be calculated for all cases where ARBs or not is not blank

• If actual daily dose is >100% of target dose, then only put in 100%

**Starting dose and target dose for ARBs**

|  | Starting dose (mg) | Target dose (mg) |
| --- | --- | --- |
| ARBs | | |
| Candesartan | 4 o.d. | 32 o.d. |
| Valsartan | 40 b.i.d. | 160 b.i.d. |
| Losartan | 50 o.d. | 100.o.d |
| Telmisartan | 40 o.d. | 80 o.d. |
| eprosartan | 400 o.d. | 600 o.d. |
| irbesartan | 75 o.d. | 300 o.d. |

**Mineralocorticoid/aldosterone receptor antagonists (MRAs) use or not**

**1=** no contraindication and any MRAs dose prescribed

**0=** no contraindication and MRAs are not prescribed

**Contraindications**

- Known allergic/other adverse reaction including hyperkalemia (K^+^ >5 mmol/L)
- Significant renal dysfunction; serum creatinine >221µ mol/L or 2.5 mg/dL or eGFR<30 mL/min/1.73m2

**Presence of MRA contraindications**

**1=** any contraindication for MRA

**0=** no contraindication for MRAs

**Diuretic use or not**

**1=** no contraindication and any diuretic dose prescribed

**0=** no contraindication and diuretic are not prescribed

**Contraindications to diuretics:** Drug specific adverse drug reactions.

**Presence of diuretics contraindications**

**1=** any contraindication for diuretics

**0=** no contraindication for diuretics

**Digoxin use or not** (Which includes if just documented as atrial fibrillation or chronic atrial fibrillation, but not Paroxysmal AF)

**1=** no contraindication and any digoxin prescribed

**0=** no contraindication and digoxin are not prescribed

**Contraindications to digoxin**

- Significant sinus or atrioventricular block (unless the block has been addressed with a permanent pacemaker).
- Trifascicular block if no report of permanent pacemaker
- Contraindicated in second‑ or third-degree heart block (without pacemaker),
- SVT involving accessory pathway (Wolff-Parkinson-White syndrome)
- Ventricular tachycardia or fibrillation (if patient has AICD in situ, then by itself is not a contraindication- is only a contraindication if there is documented history of VT or VF)
- VT and VF if no report of implantable cardioverter defibrillator (ICD) device
- Hypertrophic obstructive cardiomyopathy
- Cor pulmonale (acute and chronic) or constrictive pericarditis.
- Previous intolerance

**Digoxin contraindications**

**1=** any contraindication for digoxin

**0=** no contraindication for digoxin

**Digoxin use in presence or absence of atrial fibrillation (AF)**

**1=** used in presence chronic of AF

**2=** used in absence of chronic AF

**B. Standard criteria for heart failure with preserved ejection fraction (HFpEF)**

**Presence of hypertension or not**

**1**= where hypertension reported in comorbidity list

**0**= where hypertension has not been reported in comorbidity list

**Control of hypertension or not**

**1**= where systolic blood pressure (SBP) is less than 140 (sitting position) and diastolic blood pressure (DBP) less than 90 mm Hg

**0**= where SBP is > 140 or DBP > 90 mm Hg

**Angiotensin converting enzyme inhibitors (ACEIs) inhibitors use or not**

**1**= where any ACEIs has been prescribed

**0**= where any ACEIs has not been prescribed

**β –blockers use or not**

**1**= where any β –blockers has been prescribed

**0**= where any β –blockers has not been prescribed

**Angiotensin receptor blockers (ARBs) use or not**

**1=** where any ARB has been prescribed

**0=** where any ARB has not been prescribed

**Mineralocorticoid/aldosterone receptor antagonists (MRAs) use or not**

**1=** where any MRAs has been prescribed

**0=** where any MRAs has not been prescribed

**Loop diuretic (Furosemide) use or not**

**1=** where any loop diuretic has been prescribed

**0=** where any diuretic has not been prescribed

**Use of thiazide diuretics or not**

1= where any thiazide diuretics has been prescribed

0= where any thiazide diuretics has not been prescribed

2= renal function is abnormal (creatinine >221 µ mol/L) and thiazide is not used

**Digoxin use or not**

**1=** where digoxin has been prescribed

**0=** where digoxin has not been prescribed

**Presence of Atrial fibrillation (AF)**

**1=** presence of AF

**0=** no AF

**Anticoagulation in Atrial fibrillation**

**1**= patients anticoagulated in presence of AF

**0**= patients not anticoagulated in presence of AF

**2**= patients not anticoagulated in presence of contraindications or where there is clear documentation of reason or patient preference.

**Contraindications to use of any anticoagulant in AF:**

- Patients with increased risk of bleeding such as severe uncontrolled hypertension, recent gastrointestinal bleeding, genitourinary bleeding, active ulceration, or severe thrombocytopenia (platelet count <50,000).
- Patient after intracranial hemorrhage.

**Contraindications for use of Warfarin in AF**

- Same contraindication applicable for any anticoagulation.
- People non-adherence as well as lack of access to international normalized ratio (INR) monitoring.

**Contraindications for use of new oral anticoagulants**

- *Dabigatran:* If creatinine clearance <30 mL/min.
- *Rivaroxaban:* If creatinine clearance 30ml/min (as dose is 20mg once daily).

Apixaban: If creatinine clearance <25 mL/minute.
